# Effect of Electroacupuncture Intervention on Expression of CGRP, SP, COX-1, and PGE2 of Dorsal Portion of the Cervical Spinal Cord in Rats with Neck-Incision Pain

**DOI:** 10.1155/2013/294091

**Published:** 2013-09-02

**Authors:** Li-na Qiao, Jun-ying Wang, Yong-sheng Yang, Shu-ping Chen, Yong-hui Gao, Jian-liang Zhang, Jun-ling Liu

**Affiliations:** ^1^Department of Biochemistry and Molecular Biology, Institute of Acu-Moxibustion, China Academy of Chinese Medical Sciences, 16 Nanxiaojie Street, Dongzhimennei, Beijing 100700, China; ^2^Department of Physiology, Institute of Acu-Moxibustion, China Academy of Chinese Medical Sciences, 16 Nanxiaojie Street, Dongzhimennei, Beijing 100700, China

## Abstract

The present study was aimed to determine if cervicospinal substance P (SP) and its neurokinin-1 receptor (NK-1R), calcitonin gene-related peptide (CGRP), cyclooxygenase-1 (COX-1), and prostaglandin E2 (PGE2) were involved in electroacupuncture (EA) analgesia in neck-incision pain rats. EA intervention was applied to bilateral Futu (LI18), Hegu (LI4)-Neiguan (PC6), and Zusanli (ST36)-Yanglingquan (GB34) for 30 min. Cervicospinal SP and CGRP immunoactivity was detected by immunofluorescence technique, NK-1R and COX-1 protein and mRNA expression levels were determined using Western blot and real-time PCR, respectively, and PGE2 content was measured using ELISA. Outcomes indicated that EA of EA-LI18 and LI4-PC6 (not ST36-GB34) significantly suppressed neck-incision induced decrease of thermal pain threshold (*P* < 0.05). EA stimulation of LI18 and LI4-PC6 markedly inhibited neck-incision induced upregulation of SP and CGRP immunoactivity, NK-1 R and COX-1 mRNA and protein expression levels, as well as the increase of PGE2 content in the dorsal cervicospinal cord (*P* < 0.05). These findings showed that LI18 and LI4-PC6 EA stimulation-induced downregulation of SP, CGRP, NK-1R, COX-1, and PGE2 levels in the dorsal cervicospinal cord may contribute to their effects in relieving neck-incision pain. This study highlights the targets of EA intervention for reducing post-thyroid-surgery pain for the first time.

## 1. Introduction

Postoperative pain in patients undergoing thyroidectomy generally lasts for hours or days in spite of being moderate in severity and different in duration for the types of diseases [1]. In the treatment of this type of postoperative pain, oral administration of opioids (about 90% of the patients) and nonopioid adjuncts is the optimized choice currently [2]. However, these drugs may worsen anesthetics-induced nausea, vomiting, and other side effects [3] and the analgesic effect of these drugs is not always effective [4]. 

Therefore, a multimodal approach combining various analgesics with a nonpharmacological strategy is necessary for providing adequate postoperative pain control. Acupuncture, a component of traditional Chinese medicine, has been used to treat pain for millennia, and accumulating evidence indicates the efficacy of acupuncture for postoperative analgesia [5–10]. It has been demonstrated that the analgesic effect of acupuncture is related to its functions in upregulating plasma beta-endorphin level in patients undergoing colonoscopy [6], breast radical carcinoma operation [11], and in activating peripheral opioid pathway in a mouse model of postoperative pain [12]. It has been well documented from clinical practice of acupuncture analgesia and anesthesia that Futu (LI18) and Hegu (LI4)-Neiguan (PC6) are effective acupoints for thyroid surgery [13–16]. But there have been no research reports about acupuncture intervention for relieving postthyroidectomy pain till now. In the past several years, our research group investigated the spinal mechanism of electroacupuncture (EA) therapy underlying pain relief of thyroid surgery. Our findings showed that the analgesic effect of EA of Futu (LI18) and Hegu (LI4)-Neiguan (PC6) for neck-incision pain is closely associated with its actions in upregulating 5-HT 2A receptor (5-HT2AR) mRNA and protein, glial cell derived neurotrophic factor (GDNF), and its receptor GDNF family receptor alpha-1 (GFR alpha-1) gene expression [17, 18], and in downregulating expression levels of intracellular cAMP mRNA and cAMP response element binding protein (CREB) mRNA in the cervical spinal cord (C1–C4) [19]. 

 However, the pathogenesis of postoperative pain is very complex, including both peripheral and central neuronal changes, cellular and molecular activities, ectopia, sensitization of nociceptors, phenotypic switching, structural plasticity, disinhibition, and neuroinflammation [20–22]. Neuronal cells in the superficial layers of the spinal dorsal horn play an important role in the development and maintenance of hyperalgesia. Various mediators released from the central terminals of the primary sensory neurons contribute to this process. Among them, the excitatory amino acid glutamate, calcitonin gene related peptide (CGRP), substance P (SP), brain-derived neurotrophic factor (BDNF) and somatostatin, and so forth. Proinflammatory cytokines, chemokines, and so forth produced and released from nonneuronal cells (predominantly immune and glial cells) are also important mediators for persistent pain and all are capable of changing the response properties of central pain signaling neurons [23, 24]. Among the factors, SP, one of the neurotransmitters released from the primary nociceptive afferent endings in the dorsal horn of the spinal cord and postsynaptically binds to NK (1)-receptors [25]. CGRP, an important molecule in the spinal nociceptive processing and ensuring response from primary afferents in the spinal dorsal horn, and PGE2, the main product of cyclooxygenases (COX) and a crucial mediator for inflammatory pain sensitization via promoting synaptic transmission within the spinal cord dorsal horn [26], also contribute to the spinal pain processing network. Therefore, the present study was designed to observe their changes in the cervical spinal cord after neckincision and electroacupuncture (EA) intervention, so as to study the mechanism of acupuncture therapy underlying relieving thyroid surgery-induced pain. 

## 2. Material and Methods

### 2.1. Animals and Grouping

 Adult male Wistar rats (200–250 g), purchased from Beijing Union Medical College, were acclimatized to standard laboratory conditions (about 12 h alternate light-dark cycle) of our institute's environment first for a week and were given free access to standard chow pellet diet and water. All protocols were approved by the Institute of Acu-moxibustion, China Academy of Chinese Medical Sciences, and accorded to the Guidelines for Laboratory Animal Care and Use of the Chinese Ministry of Science and Technology. The rats were randomly assigned to 5 experimental groups: control, model, Futu (LI18), Hegu (LI4)-Neiguan (PC6), and Zusanli (ST36)-Yanglingquan (GB34), with 8 rats in each group. 

### 2.2. Thyroid Area Incision Surgery

 After detecting the baseline thermal pain threshold, the rat's neck-incision pain model was established by making a 1.5 cm longitudinal incision along the midline of the neck under isoflurane (1-2% in oxygen) inhalational anesthesia using a table-top Isoflurane anesthesia unit (VME, Matrix Company, USA), followed by repeated blunt dissection stimulation of the bilateral sternohyoideus around the thyroid gland regions for 5 min with a pair of forceps. The incision was then sutured in layers. 

### 2.3. Measurement of Thermal Pain Threshold

 The thermal pain threshold of the neck-incision area was measured 30 min before surgery, 4 h after surgical incision procedures and post-EA using a tail-flick apparatus (Model 37360, Tail Flick Unit, UGO Basile, Italy). The heat intensity was set to 25 units, and the cut-off time was set to 30 s to prevent tissue damage. The latency of escape was recorded automatically when the rat swiftly moved its neck from the heat source. Each rat was tested 3 times, with a 3–5 minutes' interval between tests. 

### 2.4. Electroacupuncture Stimulation

Four hours after neck-incision, under light anesthesia with isoflurane (0.5–1% in oxygen) via a nose cone of a table-top Isoflurane anesthesia unit, the rats of the 3 electroacupuncture (EA) treatment groups were administrated with EA stimulation following insertion of filiform needles (Gauge-32, made by Suzhou Acu-moxibustion Products Factory, China) into bilateral Futu (LI18), Hegu (LI4), Neiguan (PC6), Zusanli (ST36), and Yanglingquan (GB34), respectively. With reference to the descriptions about LI18 in the human body, the rat's LI18 is located between the sternal branch and the clavicular branch of the sternocleidomastoid muscle, at the middle of the sternocleidomastoid muscle and on the horizontal level of the 4th cervical vertebra. In the rat, LI4 is located between the 1st and 2nd metacarpal bones; PC6, about 3 mm to the wrist transverse stripe on the axopetal end; ST36, about 5 mm inferior to the capitulum fibulae and posterior-lateral to the hindlimb knee joint; and GB34, about 5 mm superior-lateral to ST36 [27]. 

After insertion, the needle handles were connected to a HANS EA Apparatus (Hans-100A, Jisheng Medical Technology, Co., Ltd., Nanjing, China) for stimulating the abovementioned acupoints with duration of 30 min, frequency of alternative 2 Hz and 15 Hz (2/15 Hz), and electric current strength of 1 mA for the first 15 min and 2 mA for the rest 15 min. Animals of the control group and model group were treated with the same anesthesia and other procedures but without EA stimulation. 

### 2.5. Tissue Preparation

 After the EA treatment and for immunofluorescence staining, rats were deeply anesthetized with a mixture solution of 20% urethane (420 mg/kg) and 1.5% chloralose (50 mg/kg, 1 : 2, 6 mL/kg, i.p.) and transcardiacly perfused through the ascending aorta with normal saline (250 mL), followed by 200 mL of 4% paraformaldehyde (Yili Fine Chemical Co., Ltd., Beijing, China) in 0.1 M phosphate buffer saline (PBS, pH 7.4). The upper segments of the cervical spinal cord (C2–C5) were removed and placed in the same fixative (4°C) overnight and then kept in 0.1 M sodium phosphate buffer (pH 7.4) containing 30% sucrose for 24 h. 

 For Western blot and quantitative real-time PCR analysis, the rats, deeply anesthetized with the same anesthetics mentioned above, were killed for collecting dorsal part of the cervical spinal cord (C2–C5, semisection along the longitudinal plane) on an ice plate after the EA treatment, kept in liquid nitrogen or stored in −80°C for mRNA and protein extraction later. 

### 2.6. Immunofluorescence Assay

 The spinal cord tissue was sectioned on a freezing microtome at 40 *μ*m for immunofluorescence double-labeling. Free-floating tissue sections were placed in 0.01 M PBS, washed with PBS Tween-20 (PBST) three times, incubated in 1 N HCL for 20 min and in 3% H_2_O_2_ in distilled water for 15 min, and blocked with 5% goat serum for 30 min at room temperature to block the unspecific staining, respectively. The sections were incubated with a mixture solution of primary antibodies: calcitonin-gene-related peptide (CGRP, diluted 1 : 500, C8198, Sigma, USA) and substance P (SP, 1 : 1000, Santa Cruz Biotechnology Inc., USA) for 36–48 h at 4°C, washed three times with phosphate buffered saline (PBS), and then incubated in the secondary antibodies, goat anti-rabbit IgG conjugated Alexa Fluor 561 (Invitrogen, red fluorescence, diluted 1 : 300) and goat anti-mouse IgG conjugated Alexa Fluor 488 (Invitrogen, green fluorescence, diluted 1 : 300) on a rocking bed (away from light) for 2 h under room temperature, respectively. 

 For control staining, primary antibody was omitted. The tissue sections were mounted on glass slides, washed four more times with running water, dried under room temperature and away from light, and sealed with coverslips at last. The analysis was performed using a light microscope Olympus AX70 with an objective magnification of 40x and software analySIS Pro 3.1. Visualization was performed with avidin-biotin complex method. Images of the spinal cord slices were acquired using confocal microscope (FV1000, Olympus, Japan). Laser channels used were 488 nm excitation and 561 nm excitation. And the fluorescence intensity was measured by Adobe Photoshop 6.0 (Adobe Systems, San Jose, CA, USA). 

### 2.7. Western Blotting Analysis

 Total protein was extracted from the tissue in RIPA lysis buffer (containing protease and phosphatase inhibitor mixtures (Roche)) by using a tissue homogenizer, followed by clearing tissue debris by centrifugation at 13000 rpm at 4°C for 20 min. Twenty micrograms of protein was loaded per lane and separated by 5% or 8% SDS-PAGE gel electrophoresis, then, transferred onto PVDF membranes. Blocking was carried out in 3% bovine serum albumin (BSA, Amresco, USA) solution for 30 min at room temperature. The membranes were incubated with primary antibody rabbit anti-NK-1R (diluted 1 : 2000, BS2632, Bioworld, USA), or rabbit anti-COX-1 (diluted 1 : 2000, 5153-1, Epitomics, USA) overnight at 4°C and with secondary antibody (1 : 20000 dilution of goat anti-rabbit Immunoglobulin G) conjugated to horseradish peroxidase (Jackson, ImmunoResearch Laboratories, West Grove, PA, USA) for 1 h at room temperature on the following day. Immunoblotting signal was detected by ECL (enhanced chemiluminescence) on chemiluminescent films. For densitometric analyses, the blots were scanned and quantified using TotalLab Quant analysis software (TotalLab Limited, England), and the result was expressed as the ratio of target gene immunoreactivity to *β*-actin immunoreactivity. 

### 2.8. Quantitative Real-Time PCR

 Total RNA was extracted with Trizol (CW0581, CWbio. Co. Ltd, Beijing, China), and then reversely transcribed with cDNA Synthesis Kit (CW0744, CWbio. Co. Ltd, Beijing, China). The reverse transcribed products were amplified. The primer sequences used were as follows: NK-1R: forward 5′-GAGCATCCCAACAGGACTTAT-3′, reverse 5′-ATGGTAGCGGTCAGAGGAGT-3′; COX-1: forward 5′-TCCTACATGGGATGACGAGC-3′, reverse 5′-GGTTGCGATACTGGAACTGG-3′; *β*-actin: forward 5′-GGAGATTACTGCCCTGGCTCCTA-3′, reverse 5′-GACTCATCGTACTCCTGCTTGCTG-3′. 

Quantitative real-time (QRT)-PCR was performed in 96-well plates using the QRT-PCR detection systems (AB7500, Applied Biosystem, USA). Three different biological replicates for each sample were performed. All the cDNA samples were amplified in triplicate from the same RNA preparation and the mean value was calculated. Each reaction included 2 *μ*L of cDNA, 10 *μ*L of REALSYBRMixture (2x), 0.8 *μ*L (10 *μ*mol/*μ*L) of both forward and reverse primers, and 7.2 *μ*L of PCR-grade water, equating to a final volume of 20 *μ*L. PCR was performed under the following conditions: 10 min at 95°C, followed by 40 cycles of 15 s at 95°C, and 60 s at 60°C. Then, the fluorescence acquisition after each cycle was performed. Finally, a dissociation curve was generated by increasing temperature from 65°C to 95°C in order to verify primer specificity. All samples for each reference gene were run on the same plate to avoid between-ran variations. The relative expression was calculated in accordance with the ΔΔCt method. Relative mRNA levels were expressed as 2^−ΔΔCt^ values. 

### 2.9. Enzyme-Linked Immunosorbent Assay (ELISA)

 The cervical spinal cord (C2–C5, semitransection) tissue specimen (1 g), added with 5 mL of homogenization buffer (0.1 M phosphate, pH 7.4, containing 1 mM EDTA and 10 *μ*M indomethacin), was homogenized at 5500 rpm for 20 s using a polytron homogenizer (PT1200, Switzerland), and then centrifuged at 4°C and 10,000 rpm for 10 min using a centrifuger (Eppendorf Centrifuge 5430, Germany). The supernatant (50 *μ*L) from each sample was added to a PGE2 assay plate for determining PGE2 levels by enzyme immunoassay (prostaglandin-E2-Monoclonal Enzyme immunoassay Kit, 2A-514010-96, Cayman, USA) according to the manufacture's protocols. A microplate reader (Multiskan MK3, Thermo, USA) at a wavelength of 410 nm was used to detect PGE2 contents, and data were computerized with Excel. The measurements were made in duplicate, and the results were expressed in pg/mg. 

### 2.10. Statistical Analysis

 The data collected in the present study were expressed as mean ± standard deviation (Mean ± SD) and analyzed by two-way repeated measures ANOVA, followed by post hoc test for least significant difference (LSD) to determine differences between two groups. *P* < 0.05 was considered statistically significant. 

## 3. Results 

### 3.1. Effects of EA on Thermal Pain Threshold

**Figure 1:**
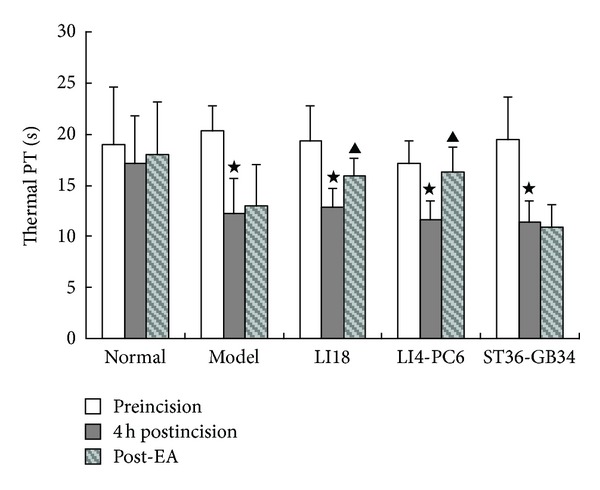
Effect of EA stimulation of Futu (LI18), Hegu (LI4)-Neiguan (PC6), and Zusanli (ST36)-Yanglingquan (GB34) on thermal pain thresholds (PT) in rats with neck incision pain (*n* = 8 in each group). Pain thresholds were measured at the time-points of pre-incision, 4 h post incision, and post-EA, and data are expressed as mean ± SD. ^⋆^ *P* < 0.05, compared with the normal group; ^▲^ *P* < 0.05, compared with the model group (after neck incision).

 Before neck-incision, the rats' thermal pain thresholds had no significant differences among the normal, model, LI18, LI4-PC6, and ST36-GB34 groups (*P* > 0.05). Four hours after the incision, the pain thresholds were significantly decreased (*P* < 0.05). Following EA stimulation of LI18 and LI4-PC6, the pain thresholds were considerably increased (*P* < 0.05), while that of EA of ST36-GB34 had no marked changes in comparison with the model group (*P* > 0.05, Figure 1). 

### 3.2. Effects of EA on the Immunoreactivity of SP and CGRP in Spinal Dorsal Horn

**Figure 2:**
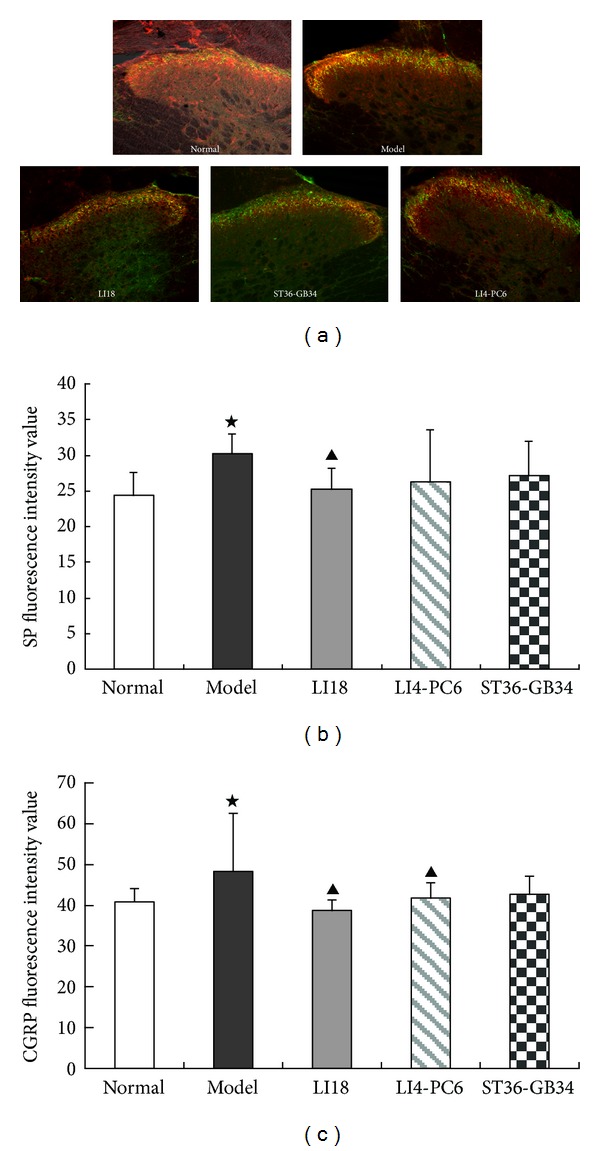
Effect of EA stimulation of different acupoints on the immunoactivity of SP and CGRP in the rat's cervical spinal cord 4 h after neck incision (*n* = 6 in each group). (a) Representative confocal microscopic photos of immunofluorescence double staining showing SP and CGRP immunoreaction (IR) positive products (green for SP and red for CGRP) in the cervical spinal cord in the 5 groups. (b) and (c) Histograms showing the mean fluorescence intensity of SP-IR positive products (b) and CGRP-IR positive products (c) in the 5 groups. Data are expressed as the mean ± SD (ANOVA, followed by LSD post hoc test); ^⋆^ *P* < 0.05, compared with the normal control group; ^▲^ *P* < 0.05, compared with the model group.

Results of immunofluorescence staining showed that SP and CGRP immunoreaction (IR)-positive products were densely distributed in the superficial layer of dorsal horns of the cervical spinal cord, particularly 4 h after neck incision (Figure 2(a)). In comparison with the normal control group, the fluorescence intensity of both SP and CGRP IR-positive products in the cervicospinal cord were significantly increased in the model group (Figures 2(b) and 2(c), *P* < 0.05), suggesting an increase of immunoactivity of SP and CGRP after neck incision. Following EA intervention, the immunoactivity levels of spinal CGRP in the LI18 group and LI4-PC6 group, and SP in the LI18 group were significantly decreased (*P* < 0.05). No apparent changes of both SP and CGRP immunoactivity were found in the ST36-GB34 group in comparison with the model group (*P* > 0.05). 

### 3.3. Effects of EA on Expression of NK-1R mRNA and Protein in Dorsal Spinal Cord

**Figure 3:**
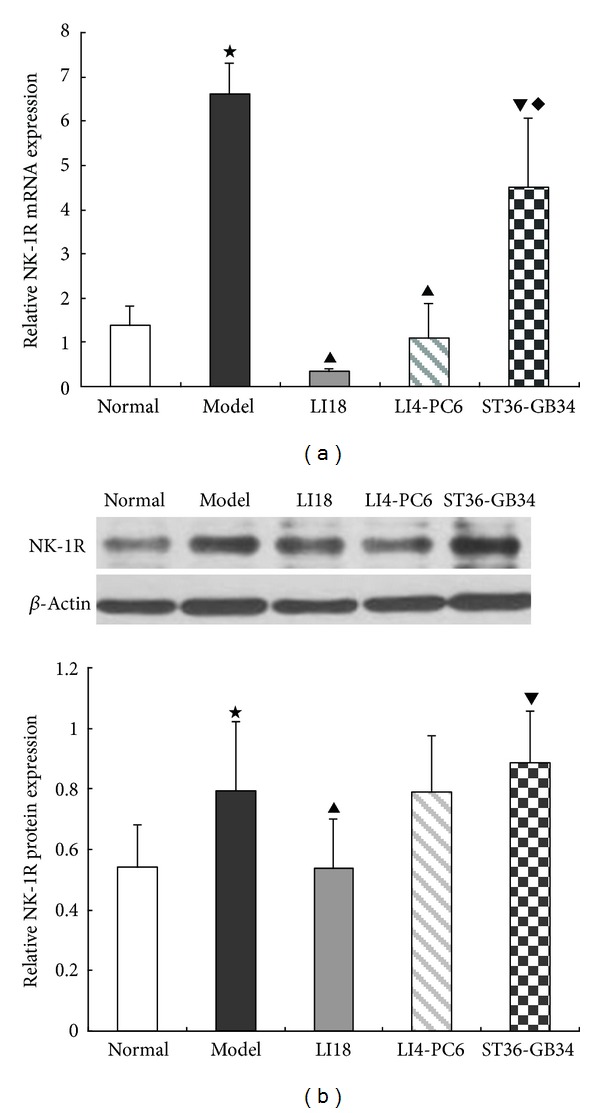
Effect of EA stimulation of different acupoints on the relative expression levels of NK-1R mRNA (a) and protein (b) in the cervical spinal cord 4 h after neck incision in rats. Data are expressed as mean ± SD (*n* = 7), (ANOVA, followed by LSD post hoc test). ^⋆^ *P* < 0.05, compared with the normal group, ^▲^ *P* < 0.05, compared with the model group; ^▾^ *P* < 0.05, compared with the LI18 group, ^*◆*^ *P* < 0.05, compared with the LI4-PC6 group.

Compared with the normal group, the relative expression levels of NK-1R mRNA and protein in the dorsal cervical spinal cord were significantly increased in the model group (*P* < 0.05, Figures 3(a) and 3(b)). Following EA intervention, the expression levels of both NK-1R mRNA and protein in the LI18 group, and NK-1R mRNA in the LI4-PC6 group were considerably lower than those in the model group (*P* < 0.05). No significant differences were found between ST36-GB34 and model groups in the expression levels of NK-1R mRNA and protein (*P* > 0.05). The effects of EA of LI18 were apparently superior to those of EA of ST36-GB34 in downregulating the expression of NK-1 mRNA and protein (*P* < 0.05). 

### 3.4. Effects of EA Intervention on Expression of COX-1 mRNA and Protein in Dorsal Spinal Cord

**Figure 4:**
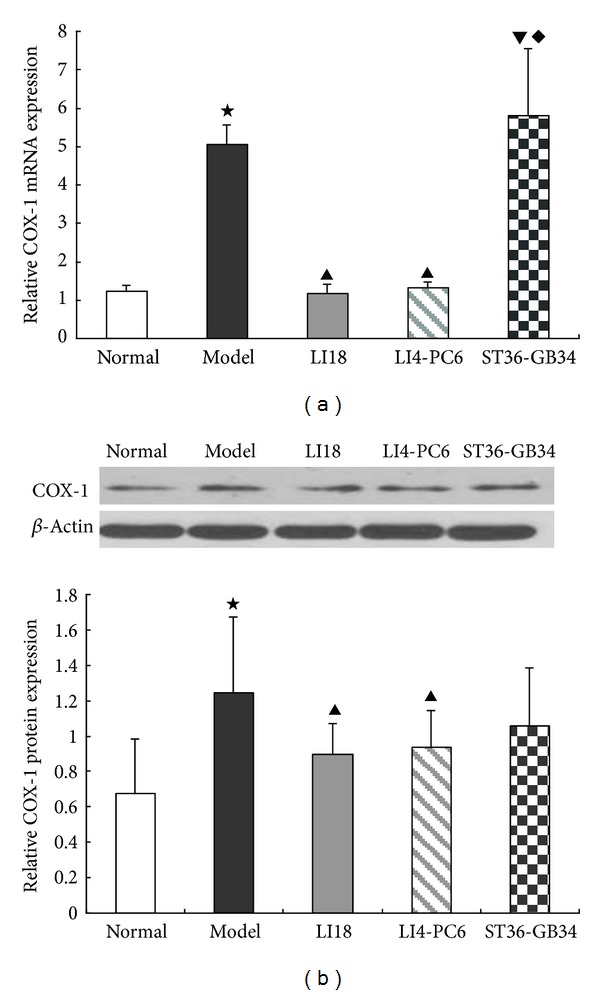
Effect of EA stimulation of different acupoints on COX-1 mRNA (a) and protein (b) expression 4 h after neck incision in rats. Data are expressed as mean ± SD (*n* = 7), (ANOVA, followed by LSD post hoc test); ^⋆^ *P* < 0.05, compared with the normal group, ^▲^ *P* < 0.05, compared with the model group; ^▾^ *P* < 0.05, compared with the LI18 group; ^*◆*^ *P* < 0.05, compared with the LI4-PC6 group.

 In comparison with the normal group, the expression levels of COX-1 mRNA and protein in the cervical spinal cord were significantly increased in the model group (*P* < 0.05, Figures 4(a) and 4(b)). After EA intervention, the expression levels of COX-1 mRNA and protein were obviously downregulated in the LI18 and LI4-PC6 groups (*P* < 0.05). No significant changes were found in COX-1 mRNA and protein expression in the ST36-GB34 group compared with the model group (*P* > 0.05). 

### 3.5. Effects of EA on PGE2 Content in the Cervical Spinal Cord

**Figure 5:**
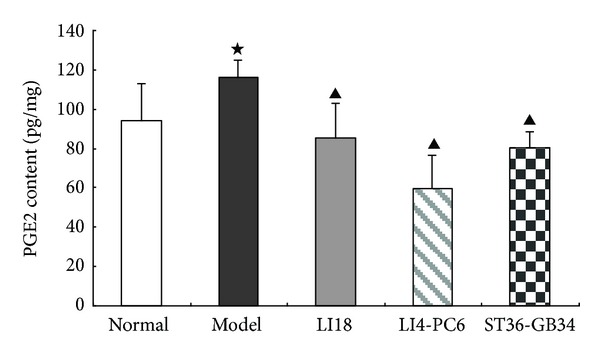
Effect of EA stimulation of different acupoints on prostaglandin E2 (PGE2) content in the dorsal spinal cord (C2–C5) 4 h after neck incision in rats. Data are expressed as mean ± SD (*n* = 8), (ANOVA, followed by LSD post hoc test). ^⋆^ *P* < 0.05, compared with the normal group, ^▲^ *P* < 0.05, compared with the model group. The level of PGE2 in the cervical spinal cord was determined using an ELISA kit.

 In comparison with the normal control group, PGE2 content of the cervical spinal cord in the model group was significantly increased 4 h after neck incision (*P* < 0.05, Figure 5). Following EA interventions of LI18 and LI4-PC6, spinal PGE2 levels in the LI18, LI4-PC6, and ST36-GB34 groups were considerably decreased (*P* < 0.05), having no significant differences among the three groups (*P* > 0.05). 

## 4. Discussion

In the present report, we characterized for the first time changes of the expression of CGRP, NK-1R, and COX-1 mRNA and proteins and PGE2 level in the cervical spinal cord in neck-incision pain rats. Results of this study showed that, 4 hours after neck incision and repeated mechanical stimulation (for miming thyroidectomy), the regional thermal pain threshold was significantly decreased. At the same time, the immunoactivity of both SP and CGRP in the superficial layers of the cervical spinal dorsal horns, the expression levels of NK-1R mRNA and protein, COX-1 mRNA and protein, and PGE2 content of the cervical dorsal spinal cord tissue were considerably increased in rats with neck-incision pain. The behavioral pain reactions, immunoactivity of SP, and the expression of NK-1R mRNA and protein and COX-1 mRNA and protein of the present study were identical to the results of our past studies using conventional immunohistochemistry in the same rat model [28] and similar study in postsurgery rats [29]. Among the indexes, spinal COX-1 expression change was the same to Zhu and colleagues' results about COX-1 immunoactivity change of the lumbar spinal cord determined by using immunohistochemistry [30] and the validated role of COX-1 in spinal hypersensitivity by intrathecal injection of preferring inhibitor, ketorolac, and the specific inhibitor SC-560 of COX-1 in paw-incision model [31]. 

It has been demonstrated that SP and CGRP coexist to a large extent (70%) in terminals of the primary afferent neurons in the dorsal horn of the spinal cord [32], and released in response to peripheral noxious stimuli [33]. Therefore, SP and CGRP (proinflammatory neuropeptides) immunoactivity was obviously increased following neck-incision and local mechanical stimulation in the present study. Simultaneously, spinal NK-1R mRNA and protein expression levels were also upregulated after neck incision. 

 Studies have showed that NK-1R is present on approximately 80% of lamina 1 neurons that project to various brain regions [34] and NK-1R on lamina I neurons activated signal transduction pathways and low-threshold (T-type) voltage-gated calcium channels synergistically and facilitated calcium-dependent long-term potentiation (LTP) at synapses from nociceptive nerve fibers. This may be the cellular mechanism of lamina I neurons in spinal dorsal horn expressing NK-1 receptor for SP mediated abnormal pain sensitivity under conditions of inflammation, trauma, or nerve injury [35]. 

Yaksh and colleagues [36] held that the postoperative hypersensitivity process results partially from a complex cascade starting with the release of SP and glutamate, followed by activation of spinal NK-1 and NMDA receptors. Among several elements, this cascade activates spinal phospholipases and generates prostanoids by COX activity, leading to spinal prostanoid release. Further studies have shown that nerve or tissue injury is associated not only with increases in NK-1 receptor density, but also with increases in NK-1 gene expression; thus, persistent activation of SP-containing primary afferent neurons might increase transcription of the NK-1 receptor mRNA and enhance the expression of NK-1 receptor protein [37], which is consistent to our observations in the present study. A cAMP response element (CRE) site occurs within the promoter region of the NK-1 receptor gene which has a CREB binding site [38]. CGRP is a transmembrane signaling molecule that increases cAMP levels and cAMP (CRE)-dependent gene expression, and also increases the levels of NK-1R mRNA in spinal neurons, indicating that CGRP regulates the expression of NK-1R via a pathway involving activation of the transcription factor, (CREB) [39]. Moreover, we have demonstrated that in formalin injection induced neck pain rats, the expression levels of cervical spinal NMDA receptor 2B subunit mRNA and its phosphorylated protein were markedly upregulated [40], but if the NMDA R is involved in neck-incision induced pain processing or not, it needs further study. 

 It was demonstrated that NK-1 receptors are not only expressed in neurons, but also in astrocytes and microglia which have a high affinity for SP, and more importantly, SP and NK-1 receptor interactions elicit activation of signal transduction pathways in both cell types and can initiate or augment pain responses [41, 42]. SP can also stimulate secretion of TNF-alpha from macrophages from which IL-1*β* was found to increase the production of SP and PGE2 in a number of neurons and glial cells [43, 44]. Prostaglandins (PGs), including PGD-2, PGE-2, PGF-2 alpha, and PGI-2 and known to be produced by cyclooxygenase (COX), catalyze their synthesis from arachidonic acid [45] and potentiate release of excitatory amino acids, SP, CGRP, and nitric oxide to enhance excitation and synaptic transmission of pain signals in the spinal cord [46]. 

COX has two forms of COX-1 and COX-2 which are constitutively expressed in the spinal cord. Double labeling studies showed that 96% of COX-1 immunoreactive cells are colocalized with microglia and 98% of COX-2 immunoreactive cells colocalized with neurons [47]. COX-1 expression was upregulated in microglia within hours after surgical incision [48–50] or thoracic muscle deep incision [51], and COX-2 played a prominent role in inflammatory pain [52–54]. These results indicate that SP, NK-1R, CGRP, COX-1, and PGE-2 in the spinal cord are all involved in postsurgery pain including neck-incision pain of the present study. Both neurons and glia cells in the cervical spinal cord are probably complicated with the neck-incision pain processing. However, the detailed interaction situations of the cervicospinal neurons and glia cells in inducing postincision pain need studying in the future. 

In addition, findings of the present study showed that following EA intervention of LI18 (at the neck) and LI4-PC6 (at the forelimb), the thermal pain thresholds were obviously upregulated, while EA stimulation of ST36-GB34 (at the hindlimb) had no apparent effect on the decreased pain threshold in neck-incision pain rats. Correspondingly, EA of LI18 and LI4-PC6 could effectively suppress neck-incision induced upregulation of expression of NK-1 mRNA, COX-1 mRNA and protein, and PGE2 content in the upper-cervical spinal cord. However, EA stimulation of ST36-GB34 had no apparent effect on SP and CGRP immunoactivity, NK-1R mRNA and protein, and COX-1 mRNA and protein expression levels in the cervical spinal cord. It suggests that the effects of EA stimulation of the three acupoints or acupoint groups in resisting pain reaction and suppressing incision-induced upregulation of spinal NK-1 mRNA and COX-1 mRNA and protein are mainly via homosegmental nerve reflex pathway. But, why the content of spinal PGE-2 was also downregulated after EA of ST36-GB34, the reason was not clear and the result needs being confirmed further. 

The results of EA analgesia and spinal SP expression are basically identical to those in our past studies [17, 28, 55] and changes of spinal PGE-2 after EA intervention are similar to those of other studies in reducing spinal PGE-2 levels in inflammatory pain rats [56, 57]. Up to now, we have not found any studies on the involvement of spinal CGRP, NK-1R mRNA and protein and COX-1 mRNA and protein in postsurgery pain. However, viewing from the known mechanisms of pain processing, we do not think that changes of these indexes are not expectable under EA analgesia. Moreover, EA analgesia might also involve its favorable regulation on the communication or interaction between neurons and glia cells in the spinal cord in neck-incision pain rats. 

 In conclusion, findings of the present study show that SP, CGRP, NK-1R, COX-1, and PGE2 in the cervicospinal cord play an important role in pain processing after neck incision in the rat. EA stimulation of LI18 and LI4-PC6 can effectively suppress neck-incision-induced sensory hypersensitivity by downregulating expression levels of spinal SP, CGRP, NK-1 mRNA, COX-1 mRNA and protein, and PGE2 content in the cervicospinal cord. These results highlight the targets of EA therapy for reducing post-thyroid-surgery pain. The exact mechanisms of EA underlying regulation of cross-talk of spinal neurons and gliacytes will be researched in the coming days. 

## References

[1] Apfelbaum JL, Chen C, Mehta SS, Gan TJ (2003). Postoperative pain experience: results from a national survey suggest postoperative pain continues to be undermanaged. *Anesthesia and Analgesia*.

[2] Ayman M, Materazzi G, Bericotti M, Rago R, Nidal YP (2003). Bupivacaine 0.5% versus ropivacaine 0.75% wound infiltration to decrease postoperative pain in total thyroidectomy, a prospective controlled study. *Minerva Chirurgica*.

[3] Barros A, Vale CP, Oliveira FC (2013). Dexamethasone effect on postoperative pain and tramadol requirement after thyroidectomy. *Pharmacology*.

[4] Ryu HR, Lee J, Park JH (2013). A comparison of postoperative pain after conventional open thyroidectomy and transaxillary single-incision robotic thyroidectomy: a prospective study. *Annals of Surgical Oncology*.

[5] Sertel S, Herrmann S, Greten HJ (2009). Additional use of acupuncture to NSAID effectively reduces post-tonsillectomy pain. *European Archives of Oto-Rhino-Laryngology*.

[6] Ni Y-F, Li J, Wang B-F (2009). Effects of electroacupuncture on bispectral index and plasma beta-endorphin in patients undergoing colonoscopy. *Zhen Ci Yan Jiu*.

[7] El-Rakshy M, Clark SC, Thompson J, Thant M (2009). Effect of intraoperative electroacupuncture on postoperative pain, analgesic requirements, nausea and sedation: a randomised controlled trial. *Acupuncture in Medicine*.

[8] Oliveira R, Prado WA (2000). Anti-hyperalgesic effect of electroacupuncture in a model of post-incisional pain in rats. *Brazilian Journal of Medical and Biological Research*.

[9] Lao L, Bergman S, Langenberg P, Wong RH, Berman B (1995). Efficacy of Chinese acupuncture on postoperative oral surgery pain. *Oral Surgery, Oral Medicine, Oral Pathology, Oral Radiology, and Endodontics*.

[10] Coura LEF, Manoel CHU, Poffo R, Bedin A, Westphal GA (2011). Randomised, controlled study of preoperative eletroacupuncture for postoperative pain control after cardiac surgery. *Acupuncture in Medicine*.

[11] Yu J-M, Qu P-S, Fan H, Wang Z, Jin Y-B, Tao F (2010). Observation on the analgesic effect of transcutaneous electrical acupoint stimulation for breast radical carcinoma operation. *Zhen Ci Yan Jiu*.

[12] Martins DF, Bobinski F, Mazzardo-Martins L (2012). Ankle joint mobilization decreases hypersensitivity by activation of peripheral opioid receptors in a mouse model of postoperative pain. *Pain Medicine*.

[13] Li YH, Ma WH, Gao XQ (2008). Comparison of the efficacy of acupuncture-assisted anesthesia with different frequency stimulation for thyroid surgery. *Guangdong Medical Journal*.

[14] Gao Y-Q, Jia Q, Yang J, Liu J-L, Shi J-H (2009). Analysis on the superiority of compound acupuncture anesthesia for thyroid ablation. *Zhen Ci Yan Jiu*.

[15] Zhang CJ, Yang F, Li M (2013). Effect of electroacupuncture combined with cervical plexus block on stress responses in patients undergoing thyroid surgery. *Zhen Ci Yan Jiu*.

[16] Wang PZ, Zhao JL, An S, Yao AH (2000). Application of local injection of anesthetics at Futu (LI18) to thyroid surgery. *Journal of Changchun College of Traditional Chinese Medicine*.

[17] Qiao L-N, Yang Y-S, Wang J-Y (2011). Effects of electroacupuncture at “Futu” (LI 18), etc. on expression of spinal 5-HT 1 AR mRNA, 5-HT 2 AR mRNA and protein in rats with neck incision pain. *Zhen Ci Yan Jiu*.

[18] Wang SJ, Tan LH, Liu JL (2012). Effect of electroacupuncture at different acupoints on expression of cervico-spinal GDNF and BDNF and their receptor genes in neck-incision pain rats. *Zhen Ci Yan Jiu*.

[19] Lin D, Kan Y, Qiao LN (2012). Effects of electroacupuncture at “Futu” (LI 18), etc. on pain threshold and cervico-spinal mGlu receptor 5/cAmp/CREB signaling in rats with neck incision pain. *Zhen Ci Yan Jiu*.

[20] Brennan TJ (2011). Pathophysiology of postoperative pain. *Pain*.

[21] Deumens R, Steyaert A, Forget P (2013). Prevention of chronic postoperative pain: cellular, molecular, and clinical insights for mechanism-based treatment approaches. *Progress in Neurobiology*.

[22] Berger JV, Knaepen L, Janssen SPM (2011). Cellular and molecular insights into neuropathy-induced pain hypersensitivity for mechanism-based treatment approaches. *Brain Research Reviews*.

[23] Pezet S, Malcangio M, McMahon SB (2002). BDNF: a neuromodulator in nociceptive pathways?. *Brain Research Reviews*.

[24] McMahon SB, Cafferty WBJ, Marchand F (2005). Immune and glial cell factors as pain mediators and modulators. *Experimental Neurology*.

[25] Sindrup SH, Graf A, Sfikas N (2006). The NK1-receptor antagonist TKA731 in painful diabetic neuropathy: a randomised, controlled trial. *European Journal of Pain*.

[26] Zeilhofer HU (2005). The glycinergic control of spinal pain processing. *Cellular and Molecular Life Sciences*.

[27] Li ZR (2003). *Experimental Acupuncturolog*.

[28] Qiao L-N, Wang J-Y, Chen S-P, Gao Y-H, Yang Y-S, Liu J-L (2010). Effects of electroacupuncture at “Futu” (LI 18) on the immunoactivity of substance P, 5-HT 1 AR, etc. of the cervical spinal dorsal horn in rats with neck incision pain. *Zhen Ci Yan Jiu*.

[29] Saxler G, Brankamp J, von Knoch M, Löer F, Hilken G, Hanesch U (2008). The density of nociceptive SP- and CGRP-immunopositive nerve fibers in the dura mater lumbalis of rats is enhanced after laminectomy, even after application of autologous fat grafts. *European Spine Journal*.

[30] Zhu X, Vincler MA, Parker R, Eisenach JC (2006). Spinal cord dynorphin expression increases, but does not drive microglial prostaglandin production or mechanical hypersensitivity after incisional surgery in rats. *Pain*.

[31] Zhu X, Conklin DR, Eisenach JC (2005). Preoperative inhibition of cyclooxygenase-1 in the spinal cord reduces postoperative pain. *Anesthesia and Analgesia*.

[32] Tuchscherer MM, Seybold VS (1989). A quantitative study of the coexistence of peptides in varicosities within the superficial laminae of the dorsal horn of the rat spinal cord. *Journal of Neuroscience*.

[33] Morton CR, Hutchison WD (1989). Release of sensory neuropeptides in the spinal cord: studies with calcitonin gene-related peptide and galanin. *Neuroscience*.

[34] Al-Khater KM, Kerr R, Todd AJ (2008). A quantitative study of spinothalamic neurons in laminae I, III and IV in lumbar and cervical segments of the rat spinal cord. *Journal of Comparative Neurology*.

[35] Ikeda H, Heinke B, Ruscheweyh R, Sandkühler J (2003). Synaptic plasticity in spinal lamina I projection neurons that mediate hyperalgesia. *Science*.

[36] Yaksh TL, Hua X-Y, Kalcheva I, Nozaki-Taguchi N, Marsala M (1999). The spinal biology in humans and animals of pain states generated by persistent small afferent input. *Proceedings of the National Academy of Sciences of the United States of America*.

[37] Taylor BK, McCarson KE (2004). Neurokinin-1 receptor gene expression in the mouse dorsal horn increases with neuropathic pain. *Journal of Pain*.

[38] Gerard NP, Garraway LA, Eddy RL (1991). Human substance P receptor (NK-1): organization of the gene, chromosome localization, and functional expression of cDNA clones. *Biochemistry*.

[39] Seybold VS, McCarson KE, Mermelstein PG, Groth RD, Abrahams LG (2003). Calcitonin gene-related peptide regulates expression of neurokinin1 receptors by rat spinal neurons. *Journal of Neuroscience*.

[40] Gao Y-H, Chen S-P, Wang J-Y, Qiao L-N, Xu Q-L, Liu J-L (2009). Effects of electroacupuncture at different acupoints on the pain behavior and NMDA receptor 2 B subunit mRNA and protein expression and phosphorylation level in the cervical spinal cord in rats with thyroid regional pain. *Zhen Ci Yan Jiu*.

[41] Marriott I (2004). The role of tachykinins in central nervous system inflammatory responses. *Frontiers in Bioscience*.

[42] Tumati S, Largent-Milnes TM, Keresztes AI (2012). Tachykinin NK1 receptor antagonist co-administration attenuates opioid withdrawal-mediated spinal microglia and astrocyte activation. *European Journal of Pharmacology*.

[43] Miyano K, Morioka N, Sugimoto T, Shiraishi S, Uezono Y, Nakata Y (2010). Activation of the neurokinin-1 receptor in rat spinal astrocytes induces Ca^2+^ release from IP_3_-sensitive Ca^2+^ stores and extracellular Ca^2+^ influx through TRPC3. *Neurochemistry International*.

[44] Jeanjean AP, Moussaoui SM, Maloteaux J-M, Laduron PM (1995). Interleukin-1*β* induces long-term increase of axonally transported opiate receptors and substance P. *Neuroscience*.

[45] Schweizer A, Feige U, Fontana A, Müller K, Dinarello CA (1988). Interleukin-1 enhances pain reflexes. Mediation through increased prostaglandin E2 levels. *Agents and Actions*.

[46] Vanegas H, Schaible H-G (2001). Prostaglandins and cycloxygenases in the spinal cord. *Progress in Neurobiology*.

[47] Ebersberger A, Grubb BD, Willingale HL, Gardiner NJ, Nebe J, Schaible H-G (1999). The intraspinal release of prostaglandin E2 in a model of acute arthritis is accompanied by an up-regulation of cyclo-oxygenase-2 in the spinal cord. *Neuroscience*.

[48] Ririe DG, Prout HM, Eisenach JC (2004). Effect of cyclooxygenase-1 inhibition in postoperative pain is developmentally regulated. *Anesthesiology*.

[49] Zhu X, Conklin D, Eisenach JC (2003). Cyclooxygenase-1 in the spinal cord plays an important role in postoperative pain. *Pain*.

[50] Prochazkova M, Dolezal T, Sliva J, Krsiak M (2006). Different patterns of spinal cyclooxygenase-1 and cyclooxygenase-2 mRNA expression in inflammatory and postoperative pain. *Basic and Clinical Pharmacology and Toxicology*.

[51] Kroin JS, Takatori M, Li J, Chen E-Y, Buvanendran A, Tuman KJ (2008). Upregulation of dorsal horn microglial cyclooxygenase-1 and neuronal cyclooxygenase-2 after thoracic deep muscle incisions in the rat. *Anesthesia and Analgesia*.

[52] Wang J-J, Chen G-J, Chen W, Du J, Luo A-L, Huang Y-G (2012). Analgesic effect of calpain inhibitor ALLN on the zymosan-induced paw inflammatory pain and its effect on the expression of cyclooxygenase-2 in the spinal dorsal horn. *Zhongguo Yi Xue Ke Xue Yuan Xue Bao*.

[53] Jeong HJ, Lee SH, Cho SY (2011). Roles of serotonergic and adrenergic receptors in the antinociception of selective cyclooxygenase-2 inhibitor in the rat spinal cord. *Korean Journal of Pain*.

[54] Yaksh TL, Dirig DM, Conway CM, Svensson C, Luo ZD, Isakson PC (2001). The acute antihyperalgesic action of nonsteroidal, anti-inflammatory drugs and release of spinal prostaglandin E2 is mediated by the inhibition of constitutive spinal cyclooxygenase-2 (COX-2) but not COX-1. *Journal of Neuroscience*.

[55] Gao YH, Chen SP, Wang JY (2012). Effects of electroacupuncture of “Futu” (LI 18), etc. on pain behavior and expression of GABA receptor subunit genes in cervical spinal cord in rats with thyroid regional pain. *Zhen Ci Yan Jiu*.

[56] Lee J-H, Jang K-J, Lee Y-T, Choi Y-H, Choi B-T (2006). Electroacupuncture inhibits inflammatory edema and hyperalgesia through regulation of cyclooxygenase synthesis in both peripheral and central nociceptive sites. *The American Journal of Chinese Medicine*.

[57] Mi W-L, Mao-Ying Q-L, Liu Q, Wang X-W, Wang Y-Q, Wu G-C (2008). Synergistic anti-hyperalgesia of electroacupuncture and low dose of celecoxib in monoarthritic rats: involvement of the cyclooxygenase activity in the spinal cord. *Brain Research Bulletin*.

